# Hypoxia-Preconditioned Wharton's Jelly-Derived Mesenchymal Stem Cells Mitigate Stress-Induced Apoptosis and Ameliorate Human Islet Survival and Function in Direct Contact Coculture System

**DOI:** 10.1155/2020/8857457

**Published:** 2020-12-17

**Authors:** Somayeh Keshtkar, Maryam Kaviani, Zahra Jabbarpour, Fatemeh Sabet Sarvestani, Mohammad Hossein Ghahremani, Elaheh Esfandiari, Mahdokht Hossein Aghdaei, Saman Nikeghbalian, Alireza Shamsaeefar, Bita Geramizadeh, Negar Azarpira

**Affiliations:** ^1^Department of Molecular Medicine, School of Advanced Technologies in Medicine, Tehran University of Medical Sciences, Tehran, Iran; ^2^Transplant Research Center, Shiraz University of Medical Sciences, Shiraz, Iran; ^3^Autophagy Research Center, Shiraz University of Medical Sciences, Shiraz, Iran; ^4^Shiraz Organ Transplant Center, Shiraz University of Medical Sciences, Shiraz, Iran

## Abstract

Protection of isolated pancreatic islets against hypoxic and oxidative damage-induced apoptosis is essential during a pretransplantation culture period. A beneficial approach to maintain viable and functional islets is the coculture period with mesenchymal stem cells (MSCs). Hypoxia preconditioning of MSCs (Hpc-MSCs) for a short time stimulates the expression and secretion of antiapoptotic, antioxidant, and prosurvival factors. The aim of the present study was to evaluate the survival and function of human islets cocultured with Hpc-MSCs. Wharton's jelly-derived MSCs were subjected to hypoxia (5% O_2_: Hpc) or normoxia (20% O_2_: Nc) for 24 hours and then cocultured with isolated human islets in direct and indirect systems. Assays of viability and apoptosis, along with the production of reactive oxygen species (ROS), hypoxia-inducible factor 1-alpha (HIF-1*α*), apoptotic pathway markers, and vascular endothelial growth factor (VEGF) in the islets, were performed. Insulin and C-peptide secretions as islet function were also evaluated. Hpc-MSCs and Nc-MSCs significantly reduced the ROS production and HIF-1*α* protein aggregation, as well as downregulation of proapoptotic proteins and upregulation of antiapoptotic marker along with increment of VEGF secretion in the cocultured islet. However, the Hpc-MSCs groups were better than Nc-MSCs cocultured islets. Hpc-MSCs in both direct and indirect coculture systems improved the islet survival, while promotion of function was only significant in the direct cocultured cells. Hpc potentiated the cytoprotective and insulinotropic effects of MSCs on human islets through reducing stressful markers, inhibiting apoptosis pathway, enhancing prosurvival factors, and promoting insulin secretion, especially in direct coculture system, suggesting the effective strategy to ameliorate the islet quality for better transplantation outcomes.

## 1. Introduction

Islet transplantation has emerged as a potential cell therapy to restore glucose homeostasis for selected type 1 diabetes patients that encounter severe hypoglycemic episodes. Despite successful results, the widespread application of islet transplantation is limited by a lack of organ donors, loss of islets during isolation, pretransplant culture period, and also after transplantation [1, 2].

Apoptosis is the most important factor contributing to the death of human islet before transplantation. The early initiator of islet destruction is hypoxia that induces apoptosis in the beta cells. Hypoxia also induces oxidative stress through reactive oxygen species (ROS) overproduction that leads to apoptosis during pre- and posttransplantation [3–7]. In clinical setting, islet culturing is performed in order to prepare the graft recipient, quality control of the cells, and transportation to other centers [8, 9]. However, studies have shown that isolated islets alone are unable to cope with hypoxia and oxidative stress during culture period. It can lead to the destruction and dysfunction of islets [3, 7, 10]. Therefore, it is necessary to improve the culture period by clinically feasible methods to inhibit apoptosis and increase the quality of islets for better transplantation outcomes.

Several studies have shown that the mesenchymal stem cells (MSCs) possess a supportive role in the survival and function of the islets during culture period and after transplantation by either secreting paracrine factors or by direct cell contact and extracellular matrix production [4, 5, 11]. Oxygen concentration is a significant factor that affects MSCs. MSCs naturally live in hypoxic niches, such as the bone marrow, adipose tissue, dental pulp, placenta, and Wharton's jelly [12–14], while these cells are usually cultured in 21% O_2_ and then cocultured with other cells or/and transplanted in ischemic sites [15, 16]. This change in the oxygen pressure reduces MSC survival rate and their therapeutic potential. Recent studies have shown that hypoxia preconditioning of MSC (Hpc-MSCs) in 1-7% O_2_ for a short time activates the Akt and extracellular regulated kinase (ERK) signaling pathways that notably enhance the production of antiapoptotic, antioxidant, prosurvival, and proangiogenic factors such as B cell lymphoma 2 (Bcl-2), vascular endothelial growth factor (VEGF), hepatocyte growth factor (HGF), catalase, and heme oxygenase-1 (Ho-1) [13, 17]. Transplanted Hpc-MSCs have shown effective therapeutic results in several conditions such as pulmonary fibrosis [18], myocardial infraction [19], acute kidney [20], brain [21], and liver [16, 22] injuries. However, studies on Hpc-MSCs in coculture or cotransplantation with islets are very limited and more research is required [15].

The aim of our study was to investigate whether Hpc-MSCs could reduce the stressful factors, inhibit mitochondrial apoptosis pathway, and improve human islet survival and function in direct and indirect coculture systems. We used Wharton's jelly-derived MSCs (WJ-MSCs) due to nonexpression of major histocompatibility class II (MHC-II), easier access, and higher proliferation capacity than other source-derived MSCs [23–25].

## 2. Methods

### 2.1. Human Pancreatic Islet Isolation

Isolation of human pancreatic islets was conducted according to the previously described protocols [26, 27]. Three pancreases were taken from dead brain donors (age, 48-60 years; negative for diabetes and cardiovascular diseases; hospitalization of less than 6 days; and cold ischemia time of 5-9 hours) after obtaining the written informed consent for research in accordance with the principles of Tehran University of Medical Sciences institutional ethics committee (IR.TUMS.REC.1394.1306). In brief, the pancreatic duct was distended with collagenase NB-1 (Serva, Germany, #17455) and neutral protease (Serva, Germany, #30301). Mechanical and enzymatic digestion was performed in Ricordi® chamber (Biorep Technologies, USA, #RC2-500M) containing several marbles. Islet purification was carried out in a continuous Biocoll (Biochrom, Germany, #L6155 and L6115) density gradient in a COBE 2991 cell processor (Terumo BCT, USA). The islet count and purity were judged by dithizone (Sigma, Germany, #43820) staining, and islets with more than 80% purity were applied for other experiments. The islet count was represented as islet equivalents (IEQ) = (50–100 *μ*m islets count/6) + (101–150 *μ*m islets count/1.5) + (151–200 *μ*m islets count × 1.7) + (201–250 *μ*m islets count × 3.5) + (251–300 *μ*m islets count × 6.3) + (301–350 *μ*m islets count × 10.4) + (>351 *μ*m islets count × 15.3) [26]. Isolated islets were maintained in nonadherent Corning™ cell culture flasks (Thermo Fisher Scientific, Canada, #353133) and cultured in RPMI 1640 (Gibco, Germany, #11875-085) medium containing 1% FBS (Gibco, UK, #10270098), 1% L-glutamine (Sigma, Germany, #G3126), 1% antibiotic/antimycotic (Sigma, Germany, #A5955), and 6.25 *μ*g/ml ITS (Sigma, Germany, #13146) in 5% CO_2_ at 37°C overnight [28], and then used for coculture experiment.

### 2.2. MSC Isolation and Characterization

Human primary MSCs were isolated from the umbilical cords collected from cesarean section delivery mothers that gave their informed consent in accordance with the Shiraz University of Medical Sciences institutional ethics committee (IR.SUMS.REC.1399.899). MSC isolation was done as previously described [29, 30]. Briefly, the small pieces of Wharton's jelly were plated in DMEM-F12 (Gibco, Germany, #12634028) supplemented with 10% FBS and 1% antibiotic/antimycotic and incubated in 5% CO_2_ at 37°C. Once the cells reached 70-80%, they were subcultured by 0.25% trypsin-EDTA (Gibco, Germany, #11560626). MSCs from passage 3 were used for characterization and other experiments.

Isolated cells were evaluated for specific surface markers CD34 (BioLegend, USA, #343506), CD45 (Dako, USA, #F0861), human leukocyte antigen (HLA-DR) (BioLegend, USA, #307632), CD44 (BioLegend, USA, #338804), CD90 (BioLegend, USA, #328108), CD73 (BioLegend, USA, #344016), CD29 (BioLegend, USA, #303004), and CD105 (BioLegend, USA, #323204) by FACS Calibur flow cytometer (Becton Dickinson, USA). Differentiation capacity of WJ-MSCs was surveyed for adipocyte (Gibco, Germany, #A1007001) and osteocyte (Gibco, Germany, #A1007201) formation with differentiation kit that stained with Oil Red O (Sigma, Germany, #O0625) and Alizarin Red S (Sigma, Germany, #A5533), respectively [31, 32].

### 2.3. Hypoxia Preconditioning of MSCs

MSCs were seeded at 20000 cells per cm^2^ in a 6-well plate (SPL Life Sciences, Korea, #30006) in RPMI 1640 containing 10% FBS and 1% antibiotic/antimycotic and incubated in 5% CO_2_ at 37°C overnight. Then, the medium was removed and fresh medium was added. The cells were placed in a hypoxia incubator chamber (Stem Cell Technologies, Canada, #27310) containing 5% O_2_, 5% CO_2_, and balanced nitrogen at 37°C for 24 hours. MSCs under normoxia condition (Nc-MSCs), 20% O_2_ and 5% CO_2_, and balanced nitrogen at 37°C were used for comparison with Hpc-WJ-MSCs.

### 2.4. Coculture of Human Islets with Hpc-MSCs and Nc-MSCs

Direct and indirect coculture systems were used in the study. For direct coculture, 1000 IEQ were added to 2 × 10^5^ cultured Hpc-MSCs and Nc-MSCs in the 6-well plates (SPL Life Sciences, Korea, #30006). For indirect coculture, the islets were placed in Transwell® insert with 0.4 *μ*m pore size (Corning, Germany, #3412); then, they were inserted on the top layer of the 6-well plates (SPL Life Sciences, Korea, #30006) that Hpc-MSCs and Nc-MSCs already cultured in them [33]. The islets alone were considered as the control group. All five groups were incubated in RPMI 1640 medium containing 10% FBS and 1% antibiotic/antimycotic for 5 days in 5% CO_2_ at 37°C.

### 2.5. Viability Assay

To evaluate the viability of the islets, we used fluorescein diacetate (Sigma, Germany, #F7378) and propidium iodide (Sigma, Germany, #P4170) for staining of live and dead cells, respectively. For the direct cocultured group, both islet and MSCs were stained. A fluorescence microscope (CKX53, Olympus, Japan) was recruited for imaging. The viability rate was reported by the percentage of live cells in the islets [3, 7, 34, 35].

### 2.6. Apoptosis Assay

To assess apoptosis in the islets, we performed terminal deoxynucleotidyl transferase-mediated dUTP nick end labeling (TUNEL) assay using Click-iT® Plus TUNEL Assay Kit (Life Technologies, France, #C10617) based on the manufacturer's protocol. After 5 days of coculture, the islets in all groups were fixed in 4% paraformaldehyde (Sigma, Germany, #P6148) and embedded in low melt agar (Sigma, Germany, #A1296). For the direct cocultured group, both islet and MSCs were fixed and separated from the plate with a scraper. After preparing 5 *μ*m sections and deparaffinization, TUNEL assay was performed. Next, DAPI (Sigma, Germany, #D9542) solution was used for nucleus staining. Imaging was done by fluorescent microscopy. The percentage of apoptotic islets was calculated based on the ratio between the TUNEL-positive cells to the nucleuses [26].

### 2.7. Gene Expression Evaluation by Real-Time PCR

To investigate the gene expression, after 5 days of coculture, RNA extraction was performed in each of the islet groups by TRIzol® reagent (Life Technologies, France, #1596026). For the direct cocultured groups, the location of islets was labeled by highlighter under the microscope; then, they were separated from MSCs with a scraper and used for RNA extraction. RNA integrity was verified by electrophoresis. RNA purification was evaluated via the Abs260 nm/Abs280 nm absorption ratio > 1.9, and 500 ng RNA was used for cDNA synthesis. Next, cDNA was synthetized with PrimeScript^TM^ RT Reagent Kit (Takara, Japan, #RR037A). We designed the following primers by NCBI tool Primer BLAST (Table 1). Ultimately, the cDNA was subjected to 40 cycles of amplification with SYBR® Premix Ex Taq^TM^ II Kit (Takara, Japan, #RR820L), using Applied Biosystems StepOnePlus™ Real-Time PCR. The fold changes were calculated by 2^−*ΔΔ*CT^ for each gene. GAPDH was used as a housekeeping gene [3].

### 2.8. Protein Expression Assay

To evaluate the protein expression, immunocytochemistry (ICC) technique was applied. After 5 days of coculture, the islets in all groups were fixed in 4% paraformaldehyde (Gibco, Germany) and embedded in low melt agar (Sigma, Germany). In the direct cocultured group, both islet and MSCs were fixed and separated from the plate with a scraper. After preparing 5 *μ*m sections and deparaffinization, the slides were subjected to ICC using primary antibodies against Bcl-2 (Abcam, France, #ab115807), Bax (Abcam, France, #ab69643), active caspase-3 (Abcam, France, #ab32042), HIF-1*α* (Medaysis, USA, #RM0374), and p53 (Dako, USA, #M7001). HRP-secondary antibody (Abcam, France, #ab6717) was used for detection after overnight incubation with primary antibodies. Positive cells for protein expression appeared with 3,3′-diaminobenzidine (Sigma, Germany, #D12384) staining and counterstained by hematoxylin (Sigma, Germany, #H3136). The protein expression rate was calculated by the H-score method using the following formula: H − score = 1 × (%mild staining) + 2 × (%moderate staining) + 3 × (%strong staining) [3, 28].

### 2.9. Glucose-Stimulated C-Peptide and Insulin Secretion

To investigate the C-peptide (Monobind, USA, #2725300A) and insulin (Monobind, USA, #5825300A) secretion in the cultured islets after 5 days, we used ELISA kit. The islets were incubated for 1 hour with RPMI 1640 without glucose (Gibco, Germany, #11879020) containing 0.5% BSA (Sigma, Germany, #A9418) and 2.8 or 20 mM glucose (Sigma, Germany, #G2354). Supernatants were collected and analyzed with ELISA reader (BioTek, China, #ELX50/80). The stimulation indexes were calculated by dividing the value of C-peptide and/or insulin secretion in 20 mM glucose medium by the value obtained upon 2.8 mM glucose medium [3, 36].

### 2.10. ROS Measurement

To evaluate the production of ROS, 2′,7′-dichlorofluorescein diacetate (DCFH-DA) (Sigma, Germany, #D6883) was used. After 5 days of coculture, the cells were placed in serum-free RPMI 1640 and 10 *μ*M DCFH-DA for 30 minutes in darkness. The fluorescence intensity was detected with a microplate reader (FLUOstar Omega®, BMG Labtech, Germany) and normalized with total protein measured by Bradford reagent (Thermo Fisher Scientific, USA, #23236) [37].

### 2.11. VEGF Secretion Measurement

After Hpc for 24 hours, the condition media of Hpc-MSCs and Nc-MSCs were collected and VEGF secretion was measured with human VEGF ELISA kit (Life Technologies, France, #KHG0111) based on the manufacturer's instruction. VEGF secretion was also measured in the Hpc-MSCs, Nc-MSCs, and islets after the 5 days of culture period. The results were represented as pg/ml.

### 2.12. Statistical Analysis

All experiments were repeated for a minimum of three times. The results were expressed as the mean ± SD. Comparison between the groups was made by unpaired Student's *t*-test for 2 groups and one-way ANOVA for multiple groups. Sidak as the post hoc test was applied for comparison between the control group and each cocultured group. The graphs were drawn in GraphPad Prism software (Version 6, San Diego, California). *P* < 0.05 was considered as statistically significant.

## 3. Result

### 3.1. Phenotypic Characterization and Differentiation of MSCs

The MSCs were positive for surface markers CD73, CD105, CD29, CD44, and CD90 and negative for surface markers CD34, CD45, and HLA-DR (Supplementary Figure S1 (A)). MSCs were differentiated into adipocyte after 14 days and osteocyte after 21 days in the culture period. Presence of intracellular lipid vacuoles in the adipocyte and calcium deposits in the osteocyte was observed (Supplementary Figure S1 (B)).

### 3.2. Comparison of VEGF Secretion in Hpc-MSCs and Nc-MSCs

**Figure 1:**
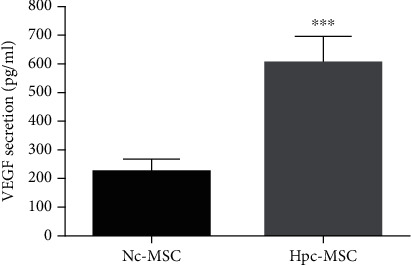
Evaluation of VEGF secretion in Hpc-MSCs compared to Nc-MSCs after 24 hours in hypoxic and normoxia condition. Hypoxia preconditioning increased the VEGF secretion in WJ-MSCs. ^∗∗^*P* < 0.01. WJ-MSCs: Wharton's jelly-derived mesenchymal stem cells; Nc-MSCs: normoxia condition MSCs; Hpc-MSCs: hypoxia preconditioning MSCs.

To confirm the positive effect of hypoxia on WJ-MSCs, we first evaluated VEGF secretion (as one of the growth factors enhanced by hypoxia) after 24 hours of culturing under hypoxic and normal condition. Results showed that the amount of VEGF in Hpc-MSCs was almost 3 times more than Nc-MSCs (606.4 ± 44.27 versus 221.5 ± 22.63) (Figure 1).

### 3.3. WJ-MSCs Increased Human Islet Survival during the Culture Period

**Figure 2:**
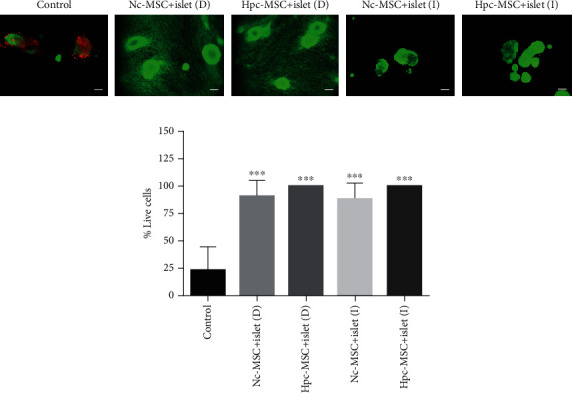
Human islet viability in coculture with Hpc-MSCs and Nc-MSCs. (a) Islet survival staining was done by fluorescein diacetate (green) for living cells and propidium iodide (red) for dead cells. (b) The charts show the viability of the islet. A comparison was done between the control and each cocultured islets. Scale bar: 100 *μ*m, ^∗∗∗^*P* < 0.001. Nc-MSCs: normoxia condition mesenchymal stem cells; Hpc-MSCs: hypoxia preconditioning MSCs; D: direct coculture; I: indirect coculture.

Survival assessment showed that the percentage of the viable islet in the control group was approximately 25% during the culture period, and coculture with MSCs increased the islet viability to 100%. Survival rate was similar in the Hpc-MSCs and Nc-MSCs cocultured groups, and there was no significant difference between them (Figure 2).

### 3.4. WJ-MSCs Inhibited the Human Islet Apoptosis during the Culture Period

**Figure 3:**
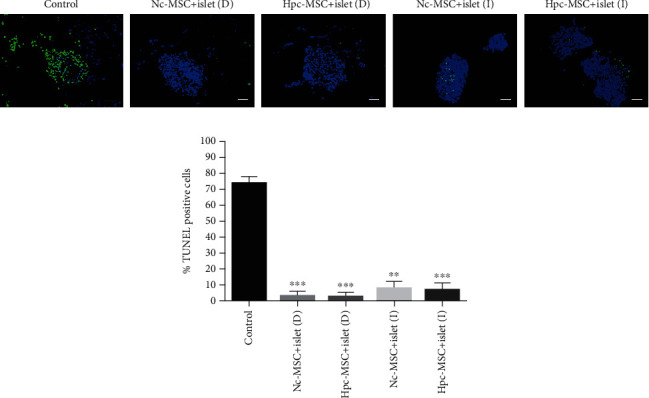
Human islet apoptosis in coculture with Hpc-MSCs and Nc-MSCs. (a) Apoptotic islets were green florescent, and stained nuclei were blue by DAPI dye. (b) The charts show the percentage of TUNEL-positive islets. A comparison was done between the control and each cocultured islets. Scale bar: 60 *μ*m. ^∗^*P* < 0.05 and ^∗∗∗^*P* < 0.001. Nc-MSCs: normoxia condition mesenchymal stem cells; Hpc-MSCs: hypoxia preconditioning MSCs; D: direct coculture; I: indirect coculture.

The percentage of apoptotic islets was about 70% in the control group, whereas coculture with WJ-MSCs decreased the islet death to less than 10%. The reduction in the two direct coculture groups was more significant than the indirect groups, but there was no difference between the Hpc-MSCs and Nc-MSCs groups (Figure 3).

### 3.5. Increment of Bcl-2 and Reduction of Bax and Caspase-3 in the Cocultured Islet

**Figure 4:**
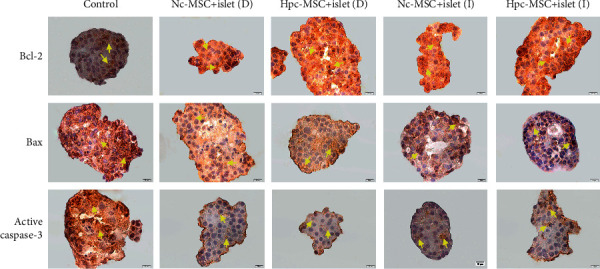
Immunocytochemistry of Bcl-2, Bax, and active caspase-3 in human islets cocultured with WJ-MSCs. The significant enhancement of Bcl-2 and reduction of Bax and active caspase-3 protein levels in the cocultured islets with Hpc-MSCs and Nc-MSCs (yellow arrows show positive protein expression). Scale bar: 10 *μ*m. WJ-MSCs: Wharton's jelly-derived mesenchymal stem cells; Nc-MSCs: normoxia condition MSCs; Hpc-MSCs: hypoxia preconditioning MSCs; D: direct coculture; I: indirect coculture.

**Figure 5:**
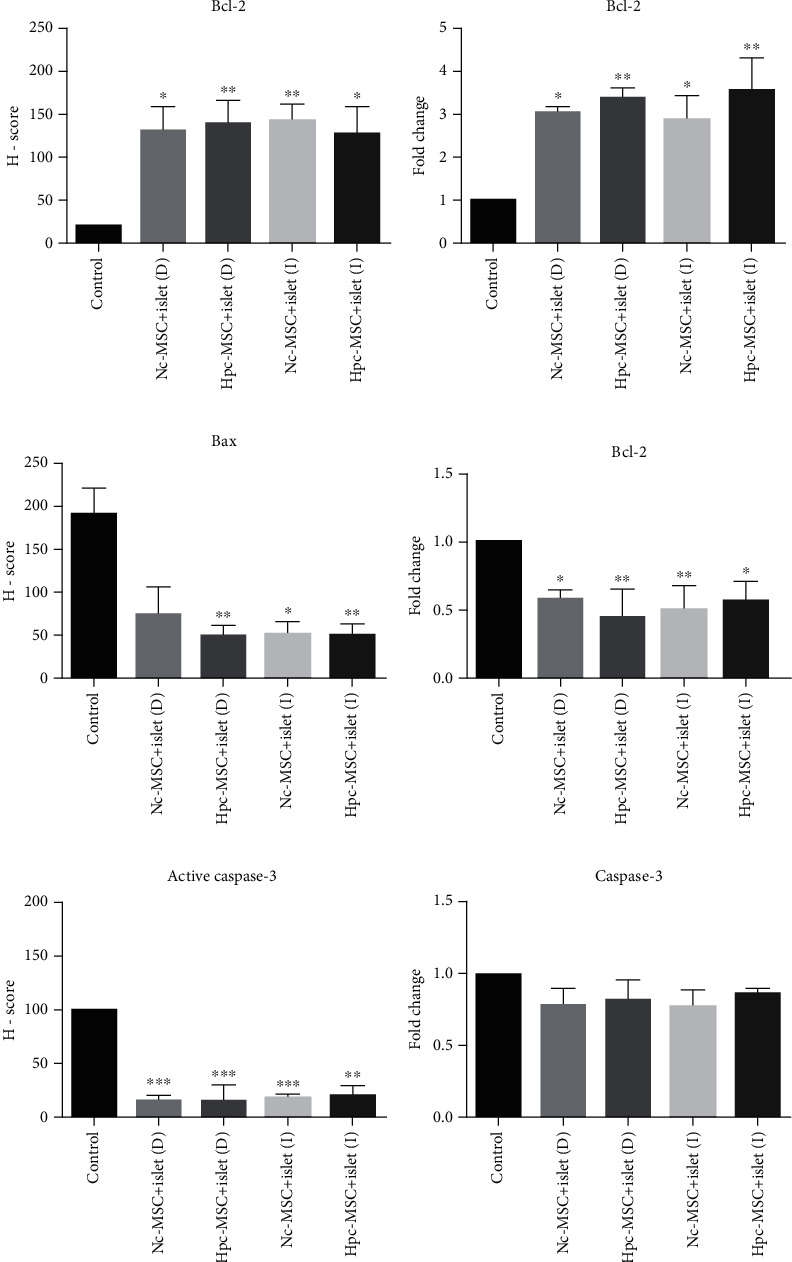
Protein and gene expression of Bcl-2, Bax, and caspase-3 in human islets cocultured with Hpc-MSCs and Nc-MSCs. (a–c) Bax, Bcl-2, and active caspase-3 protein in the islets expressed as graphs based on the H-score method. (d–f) Bax, Bcl-2, and caspase-3 mRNA expression in the islets. A comparison was done between the control and each cocultured islets. ^∗^*P* < 0.05, ^∗∗^*P* < 0.01, and ^∗∗∗^*P* < 0.001. Nc-MSCs: normoxia condition mesenchymal stem cells; Hpc-MSCs: hypoxia preconditioning MSCs; D: direct coculture; I: indirect coculture.

The antiapoptotic marker of Bcl-2 significantly increased, and proapoptotic marker of Bax significantly decreased at gene expression and protein level in the cocultured islets (Figures 4 and 5(a)–5(d)). There was no obvious difference between the Hpc-MSCs and Nc-MSCs groups.

Active caspase-3 protein as a main indicator of apoptosis was significantly mitigated in the cocultured islets. Gene expression of caspase-3 was also decreased in all cocultured groups; however, it was not significant in any groups (Figures 4 and 5(e) and 5(f)).

### 3.6. The Direct Cocultured Hpc-MSCs Group Significantly Increased the Islet Function

**Figure 6:**
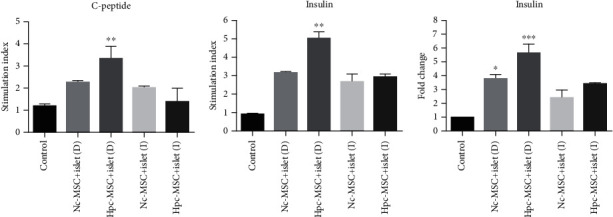
Glucose stimulation of (a) C-peptide and (b) insulin secretions and (c) insulin mRNA expression in human islets cocultured with Hpc-MSCs and Nc-MSCs. A comparison was done between the control and each cocultured islets. ^∗^*P* < 0.05, ^∗∗^*P* < 0.01, and ^∗∗∗^*P* < 0.001. Nc-MSCs: normoxia condition mesenchymal stem cells; Hpc-MSCs: hypoxia preconditioning MSCs; D: direct coculture; I: indirect coculture.

Insulin and C-peptide secretion was increased in all cocultured islets. The increment was greater in the direct cocultured groups than indirect ones. However, the significant increase was only observed in the direct cocultured Hpc-MSCs group (Figures 6(a) and 6(b)). The insulin mRNA level was enhanced in all cocultured groups. Statistical significance was seen in both the direct cocultured Hpc and Nc-MSCs groups (Figure 6(c)).

### 3.7. HIF-1*α* Was Detected in the Islets and Decreased in the Cocultured Groups

**Figure 7:**
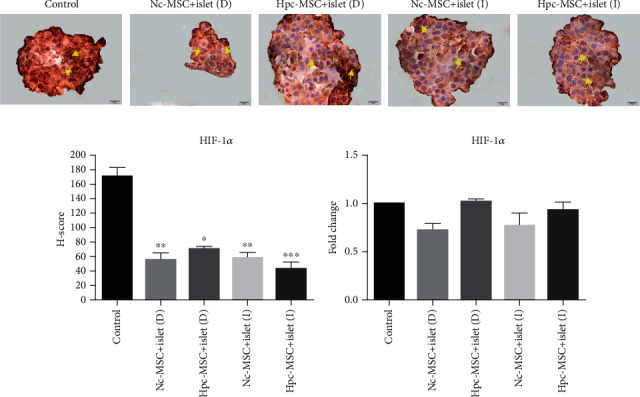
HIF-1*α* protein and mRNA expression in human islets cocultured with Hpc-MSCs and Nc-MSCs. (a) HIF-1*α* protein immunocytochemistry. (b) HIF-1*α* protein level in the islets expressed as graphs based on the H-score method. (c) HIF-1*α* gene expression in the islets. A comparison was done between the control and each cocultured islets (yellow arrows show positive protein expression). Scale bar: 10 *μ*m. ^∗^*P* < 0.05 and ^∗∗^*P* < 0.01. Nc-MSCs: normoxia condition mesenchymal stem cells; Hpc-MSCs: hypoxia preconditioning MSCs; D: direct coculture; I: indirect coculture.

HIF-1*α* protein as a main hypoxia marker that stabilized under hypoxic condition was expressed in all groups, but it reduced in the presence of WJ-MSCs in the cocultured islets (Figures 7(a) and 7(b)). HIF-1*α* mRNA expression reduced in the islet cocultured groups compared to the control group, but the decrement was not significant. It may be related to regulation of HIF-1*α* at protein stability level under hypoxia [3] (Figure 7(c)).

### 3.8. The Suppression of p53 Protein in the Presence of WJ-MSCs

**Figure 8:**
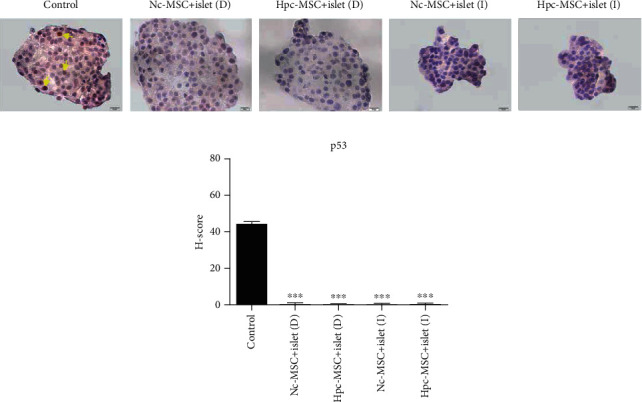
p53 protein level in human islets cocultured with Hpc-MSCs and Nc-MSCs. (a) p53 protein immunocytochemistry. (b) p53 protein level in the islets as graphs based on the H-score method. A comparison was done between the control and each cocultured islets (yellow arrows show positive protein expression). Scale bar: 10 *μ*m. ^∗^*P* < 0.05, ^∗∗^*P* < 0.01, and ^∗∗∗^*P* < 0.001. Nc-MSCs: normoxia condition mesenchymal stem cells; Hpc-MSCs: hypoxia preconditioning MSCs; D: direct coculture; I: indirect coculture.

p53 protein was detected as one of the downstream targets of HIF-1*α* that stabilized during prolonged hypoxia condition. The results showed that p53 was only present in the control group and inhibited in all cocultured islets, suggesting the antiapoptotic effect of WJ-MSCs on islet apoptosis (Figure 8).

### 3.9. The Significant Reduction of ROS in the Presence of WJ-MSCs

**Figure 9:**
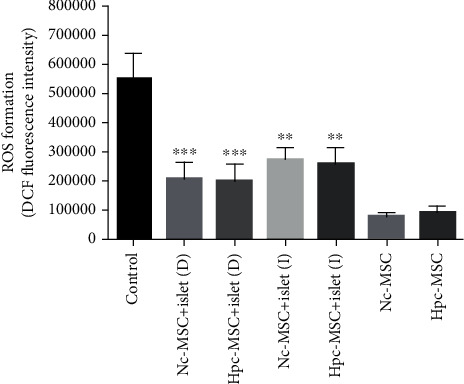
ROS production in human islets cocultured with Hpc-MSCs and Nc-MSCs. The chart shows ROS formation based on DCF fluorescent intensity. A comparison was done between the control and each cocultured islets. ^∗∗^*P* < 0.01 and ^∗∗∗^*P* < 0.001. Nc-MSCs: normoxia condition mesenchymal stem cells; Hpc-MSCs: hypoxia preconditioning MSCs; D: direct coculture; I: indirect coculture.

Production of ROS as an oxidative marker that was related to induction of apoptosis in the isolated islets was measured. The amount of ROS in Nc-MSCs and Hpc-MSCs alone was also detected for normalization of the cocultured groups after finishing the culture period. Comparison was performed between the islet control group and cocultured groups (Figure 9). The ROS production was the highest in the islet control group, while it was lower in the islet indirectly cocultured with Hpc-MSCs and Nc-MSCs. In the presence of contacting MSC, production of ROS was the lowest among all islet groups. It might indicate the antioxidant effect of MSCs on the isolated islets. Also, the amount of ROS in Nc-MSCs (control) was lower than Hpc-MSCs (control) after 5 days of culture period, but the difference was not significant.

### 3.10. VEGF Significantly Enhanced in the Cocultured Islets with Hpc-WJ-MSCs

**Figure 10:**
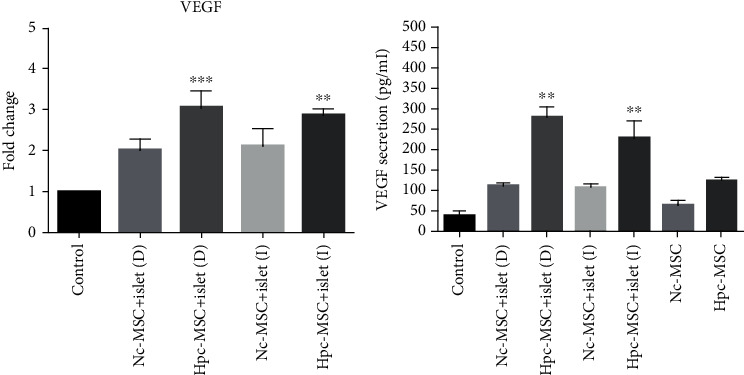
VEGF secretion and mRNA expression in human islets cocultured with Hpc-MSCs and Nc-MSCs. (a) VEGF mRNA expression and (b) VEGF secretion in the islet groups. A comparison was done between the control and each cocultured islets. ^∗∗^*P* < 0.01 and ^∗∗∗^*P* < 0.001. Nc-MSCs: normoxia condition mesenchymal stem cells; Hpc-MSCs: hypoxia preconditioning MSCs; D: direct coculture; I: indirect coculture.

VEGF mRNA was the lowest in the islet control group, whereas it induced transcription at relatively high level in the islets cocultured with Hpc-MSCs and Nc-MSCs in direct and indirect contact. However, it was just significantly seen in the direct and indirect Hpc-MSCs cocultured groups (Figure 10(a)).

VEGF secretion was also evaluated in the islet control group and islet cocultured groups. The amount of VEGF secretion in the Nc-MSCs and Hpc-MSCs after 5 days of culture accounted for normalization of the secretion of VEGF in the cocultured groups. Comparison was made between the islet control and cocultured groups (Figure 10(b)). VEGF secretion was the highest in the HPc-MSCs coculture groups (direct and indirect), intermediate in the Nc-MSCs coculture groups, and the lowest in the islet control group. Interestingly, the amount of VEGF in Hpc-MSCs alone remained higher than Nc-MSCs after 5 days of culture period.

## 4. Discussion

Pretransplant culture of the pancreatic islet is an inevitable step in preparing the transplant-recipient diabetic patient and also evaluating the quality of the isolated islets [8, 9]. However, isolated islets experience hypoxia and oxidative stress during the culture period that induces cell death through apoptosis pathway with subsequent decrement in the survival rate and impairment of function [3, 7, 38].

One of the practical strategies for protection of isolated islets against apoptosis is coculture with MSCs [5]. It has also been proven that Hpc for 24-72 hours enhances the proliferation and survival capacity of MSCs for a longer time [12]. Hpc increases the regenerative and cytoprotective effects of MSCs through increment of phosphorylation of Akt and ERK, enhancement of Bcl-2 expression, suppression of active caspase-3, and upregulation of secretion factors such as VEGF, HGF, fibroblast growth factors, and insulin-like growth factor [13, 15]. In agreement with these documents, the significant increment of VEGF secretion was observed in Hpc-WJ-MSCs relative to Nc-WJ-MSCs in our study. Several studies have reported that the Hpc-MSCs significantly improved the cell function and recovered the tissues compared with Nc-MSCs in both in vitro and in vivo models [16, 18–22].

The present study showed that the quality of islets in the control group declined and the cells experienced widespread mortality. Coculture of islets with Hpc-MSCs and Nc-MSCs significantly enhanced the viability rate, reduced the apoptosis cells, and downregulated Bax and active caspase-3 along with upregulation of Bcl-2. Nevertheless, improvement of the islets' survival was slightly better in the Hpc-MSCs groups compared to the Nc-MSCs groups. Our observations were in agreement with other studies on Hpc-MSCs. It was reported that direct coculture of Hpc-adipose tissue MSCs (AD-MSCs) with hepatocytes decreased the proapoptotic genes and increased the expression of Bcl-2 in the cocultured hepatocytes that led to improvement in the hepatocyte survival and metabolism. However, indirect coculture had minimal effects on the hepatocyte [16]. Likewise, it was shown that Hpc-bone marrow MSCs (BM-MSCs) enhanced the angiogenesis and neurogenesis in cerebral ischemia rat model through upregulation of growth factors including BDNF, GDNF, VEGF, and erythropoietin with downregulation of inflammatory cytokines [21]. Overall, all these studies reported that Hpc-MSCs showed a better effect compared to Nc-MSCs.

Given the importance of viable and efficient cell numbers in pancreatic islet transplantation, it is necessary to investigate the causes that induce cell death. Human islets make up less than 2% of the pancreas, but they consume about 10% of the total volume of the organ oxygen [39]. These cells have dense capillary network but are not physiologically ready for exposure to hypoxia. Hypoxia induces oxidative stress by ROS overproduction after reoxygenation [40, 41]. Besides, the islets possess a poor antioxidant defense system [42]. Therefore, the isolated islets are very vulnerable to hypoxia and oxidative conditions during the culture period [39]. HIF-1*α* can act as an antiapoptotic or proapoptotic factor depending on the cell type and severity of hypoxia [43]. When a cell is faced to mild hypoxia, HIF-1*α* transcripts a set of genes such as VEGF and acts as a prosurvival factor. However, following prolonged hypoxia, the protective adaptation of HIF-1*α* would not be sufficient and the cell switches toward apoptosis by p53 stabilization and Bax enhancement [3, 43]. In addition, overproduction of ROS induced apoptosis by either the mitochondrial membrane destruction or cytochrome C release that triggers caspase cascade and/or the stabilization of HIF-1*α* and p53 [44]. Previous research has proven that islet apoptosis occurs after intracellular hypoxia, HIF-1*α* protein aggregation, along with a ROS overproduction [10, 38, 42, 45]. In our study, the islets alone were vulnerable to hypoxia and oxidative stress conditions and shift to apoptosis via ROS overproduction parallel with higher aggregation of HIF-1*α* that was followed with p53 stabilization and apoptosis pathway activation. The expression and secreted VEGF in the control group was also not sufficient to help the islet survival. However, Hpc-MSCs significantly aid the islets to cope with this stress and inhibit apoptosis pathway in both coculture systems, suggesting antioxidant and antiapoptotic potential of Hpc-MSC on the cocultured islets. A recent study has reported that the condition media obtained from Hpc-AD-MSCs mitigated the apoptosis and improved the islet survival during the culture period. They suggested that the positive effect of Hpc-AD-MSCs was mainly due to the high production of growth factors, especially VEGF [15]. The VEGF supplement and also gene transfection have been shown to improve the islet revascularization and survival [46–48]. It was also observed that angiogenic compounds like liraglutide increased the VEGF secretion in the isolated rat islets and promoted its survival [49]. On the other hand, islet revascularization after transplantation is a critical process in which VEGF plays a significant role [38, 49]. In the present study, VEGF secretion in Hpc-WJ-MSCs was 3 times that of Nc-WJ-MSCs after 24 hours of hypoxia preconditioning. In addition, VEGF expression and secretion of the islets significantly increased in both direct and indirect cocultured Hpc-MSCs. Although we did not investigate the mechanisms under which Hpc-MSCs reduced apoptosis and improved the islet survival, the paracrine factors such as VEGF secretion and direct cell contact are suggested to be further considered.

Having functional islet is an important factor for successful transplantation. The results of the present study showed that cocultured islet, especially in the direct coculture groups, increased insulin expression and secretion. Notably, this increase was only significant in the direct Hpc-MSCs group. To justify the effect of direct coculture on the islet function, previous studies have shown that direct coculture of the islets with Nc-AD-MSCs significantly increased the insulin secretion through extracellular matrix production and the high expression of annexin A1 by MSCs [50]. Annexin A1 is a ligand activated by MSCs that binds to G protein-coupled receptors (GPCRs) of the islets and enhances glucose-stimulated insulin secretion [51]. Similarity, it was reported that Nc-BM-MSCs in direct contact with islets through the N-cadherin molecules improved the insulin secretion [52]. Adhesion molecules such as cadherin and integrin have been shown to be expressed in human islets and are involved in regulating the insulin secretion. Indeed, enhancement of the islet function in direct cell contact with MSCs requires N-cadherin interaction and its blocking prevents the increase of insulin secretion in the cocultured islets [52]. The mentioned studies reported the effect of direct cocultured Nc-MSCs on the islet function. To the best of our knowledge, the present study is the first report in which direct contact of Hpc-WJ-MSCs exerted a significant effect on insulin expression and secretion compared to Nc-MSCs, probably through more activation of the mentioned mechanisms.

There are many reports about the protective effect of BM-MSCs and AD-MSCs on the islets in normoxia condition. Some studies pointed to the positive effect through cell-to-cell contact, while others emphasized the protective effect of indirect coculture and paracrine activity [5, 50, 53, 54]. Moreover, it was reported that a coculture period of the islets with Nc-AD-MSCs improved the engraftment outcomes and achieved normoglycemia in transplanted diabetic mice model [11].

Studies have proven that WJ-MSCs due to the noninvasive process of isolation, high proliferation, unique immunomodulatory properties, and nonexpression of MHC-II are a good option for allogeneic transplantation [55]. Our study for the first time evaluated the human islets directly and indirectly cocultured with Hpc-WJ-MSCs. Based on our results, Hpc-WJ-MSCs are more effective in the protection of human islet viability and function compared to Nc-WJ-MSCs. We emphasized that Hpc-MSCs reduced ROS production, regulated HIF1*α* aggregation, suppressed p53, and enhanced VEGF secretion and in this way induced a change in the islet phenotype from proapoptotic to antiapoptotic. Besides, the increase of insulin expression and secretion was significantly observed in direct contact. Indeed, we supposed that Hpc improved antiapoptotic, antioxidant, and insulinotropic properties of MSCs. The protective effect of Hpc-WJ-MSCs against hypoxia and oxidative damage at least in part is related to VEGF secretion and direct cell contact with islets, suggesting the potential of Hpc-WJ-MSCs in improving human islet quality in the clinical transplantation. Recent study by Mohammad et al. showed that stem cells exist in adult mouse pancreas that regenerate diabetic pancreas after partial pancreatectomy [56]. They found that these stem cells firstly regenerated the exocrine compartment and later they differentiated to new islets. In other publications, infusion of MSCs in diabetic patients had beneficial clinical effects and suggested to provide protective effects via their antioxidant and antiapoptotic capacity for promotion of islet viability. Moreover, MSCs prevented the deterioration from impaired glucose tolerance to type 2 diabetes, at least in the short term [57]. In our study, according to the high mortality of islets in the control group, it seems that islets need to be protected during culture period and Hpc-MSCs notably could protect these cells with antiapoptotic, antioxidant, and insulinotropic properties. Although some studies on mesenchymal stem cells refer only to the role of their paracrine activity [56, 58], our study and other researches point to the role of paracrine activity associated with cell-to-cell interactions [5, 50, 52, 59]. Therefore, we suggested the Hpc-WJ-MSCs coculture method to overcome apoptosis and poor human islet function; both of which impede the success of transplantation. Future in vivo studies for transplantation of cocultured islet with Hpc-WJ-MSCs are required to confirm these results. Moreover, finding precise mechanisms under which Hpc-MSCs improve the islet quality is necessary.

## 5. Conclusion

Given the bright horizon drawn in application of pancreatic human islet for diabetes type 1 treatment, attention to all aspects related to the optimal isolation, culture period, and transplantation of the islets is necessary. In summary, Hpc-WJ-MSCs as the supporting cells during pretransplant culture period could protect the isolated human islets from the stress-induced apoptosis and provided more viable and functional islets for successful transplantation, especially through direct coculture system.

## Figures and Tables

**Table 1:** The designed primers.

Gene	Product (bp) size	Sequence	Annealing temperature (°C)
Glyceraldehyde 3-phosphate dehydrogenase (GAPDH)	127	F: GCTCATTTCCTGGTATGACAACG	61
		R: CTCTCTTCCTCTTGTGCTCTTG	
B cell lymphoma 2 (Bcl-2)	105	F: GATGGGATCGTTGCCTTATGC	62
		R: CAGTCTACTTCCTCTGTGATGTTGT	
BCL-2-associated X protein (Bax)	134	F: TTCTGACGGCAACTTCAACT	60
		R: GGAGGAAGTCCAATGTCCAG	
Insulin	150	F: CTTCTACACACCCAAGACCC	60
		R: CTGGTACAGCATTGTTCCAC	
Vascular endothelial growth factor (VEGF)	111	F: CTTCAAGCCATCCTGTGTGC	58
		R: ATCCGCATAATCTGCATGGTG	
Hypoxia-inducible factor 1-alpha (HIF-1*α*)	131	F: GCAGCAACGACACAGAAACT	60
		R: TTCAGCGGTGGGTAATGGAG	
Caspase-3	148	F: ACTCCACAGCACCTGGTTATT	61
		R: TCTGTTGCCACCTTTCGGTT	

## Data Availability

Data sharing is not applicable.

## Supplementary Materials

Supplementary Materials Methodological details for phenotypic characterization and differentiation of WJ-MSCs (Supplementary Materials).
