# Supplementary material for: Hypoxia-Preconditioned Wharton's Jelly-Derived Mesenchymal Stem Cells Mitigate Stress-Induced Apoptosis and Ameliorate Human Islet Survival and Function in Direct Contact Coculture System
Source: Stem Cells Int. 2020 Dec 17;2020:8857457. doi: 10.1155/2020/8857457 (PMC7759420; doi:10.1155/2020/8857457)
Supplement: Supplementary Materials — Methodological details for phenotypic characterization and differentiation of WJ-MSCs (Supplementary Materials). [file 8857457.f1.docx]

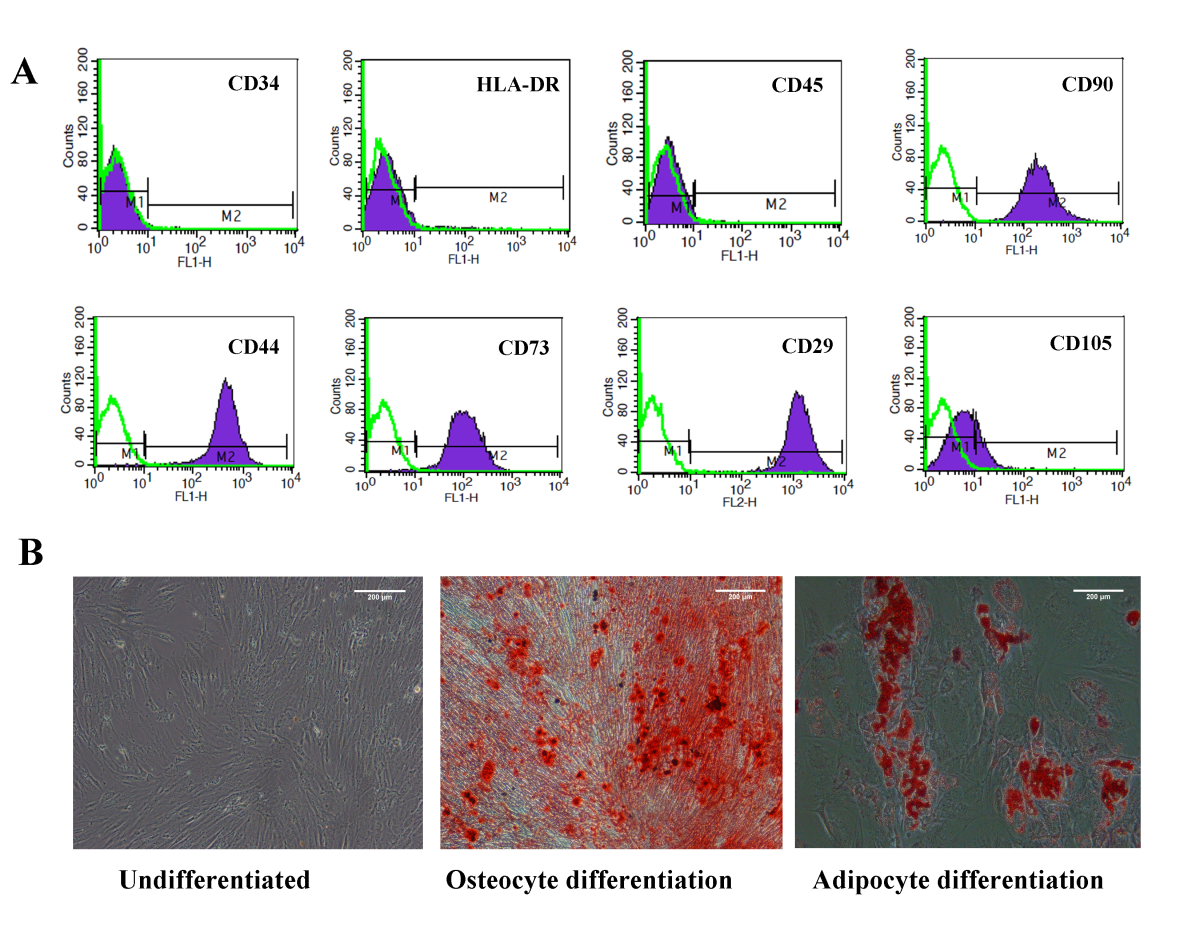


**Supplementary Figure S1.** **Phenotypic characterization and differentiation of WJ-MSCs** **(A)** Flow cytometry histogram surface of WJ-MSCs for CD 34, HLA-DR, CD45, CD 90, CD44, CD73, CD29 and CD 105 markers. Green histograms indicate the isotype control, and purple histograms indicate the signal for each specific marker. **(B)** Differentiation of WJ-MSCs into the osteocyte and adipocyte that is shown with Alizarin red S and Oil red O staining, respectively. WJ-MSCs: Wharton’s Jelly derived mesenchymal stem cells
